# Improved cell-specificity of adeno-associated viral vectors for medullary thyroid carcinoma using calcitonin gene regulatory elements

**DOI:** 10.1371/journal.pone.0228005

**Published:** 2020-02-06

**Authors:** Hazel C. Levy, Danielle Hulvey, Laura Adamson-Small, Natacha Jn-Simon, Victor Prima, Scott Rivkees, Jacqueline A. Hobbs

**Affiliations:** 1 Department of Pediatrics, College of Medicine, University of Florida, Gainesville, Florida, United States of America; 2 Department of Psychiatry, College of Medicine, University of Florida, Gainesville, Florida, United States of America; Universidade do Porto Faculdade de Medicina, PORTUGAL

## Abstract

Targeted gene therapy using recombinant adeno-associated virus (rAAV) vectors is a potential therapeutic strategy for treating cancer, and tissue-specific promoters may help with tissue targeting. Medullary thyroid carcinoma (MTC) is a disease of the calcitonin secreting thyroid C cells, and calcitonin is highly expressed in MTC tumors compared to other cells. To target MTC cells, we evaluated an rAAV serotype 2 vector (rAAV2-pM+104-GFP) containing a modified calcitonin/calcitonin gene related peptide promoter (pM+104) and a green fluorescent protein (GFP) reporter gene. *In vitro* transduction experiments comparing the MTC TT cell line with non-MTC cell lines demonstrated that rAAV2-pM+104-GFP infection yielded significantly (p < 0.05) higher GFP expression in TT cells than in non-MTC cell lines (HEK293 and HeLa), and significantly higher expression than in TT cells infected with the positive control rAAV2-pCBA-GFP vector. The rAAV2-pCBA-GFP control vector included a well-characterized, ubiquitously expresses control promoter, the chicken beta actin promoter with a cytomegalovirus enhancer (pCBA). *In vivo* experiments using a TT cell xenograft tumor mouse model showed that tumors directly injected with 2 x 10^10^ vg of rAAV2-pM+104-GFP vector resulted in GFP expression detected in 21.7% of cells, 48 hours after the injection. Furthermore, GFP expression was significantly higher for rAAV-pM+104-GFP treatments with a longer vector treatment duration and higher vector dose, with up to 52.6% (q < 0.05) GFP cells detected 72 hours after injecting 1x 10^11^ vg/tumor. These data show that we have developed an rAAV vector with improved selectivity for MTC.

## Introduction

Medullary thyroid carcinoma (MTC) originates from the calcitonin secreting parafollicular thyroid C cells [1]. Although MTCs comprise about 5% of thyroid cancers, they cause 14% of thyroid cancer-related deaths, and once metastasized there is no cure [2–4]. Hereditary and sporadic point mutations in the rearranged during transfection (RET) receptor tyrosine kinase oncogene are linked to greater than 60% of MTC cases [5]. Germline mutations in RET, seen in 98% of patients with hereditary MTC [6], are associated with several clinical conditions including familial MTC (FMTC) [7–10] multiple endocrine neoplasia (MEN) type 2A [11–13], and MEN2B [11, 12, 14].

The MTC cell line TT is the only commercially available human MTC cell line. The TT cell line is a well-characterized MTC model system. The MEN2A-type RET mutation C634R leads to ligand-independent RET dimerization, causing constitutive autophosphorylation activity that promotes malignant transformation in these cells [15].

The calcitonin/calcitonin gene related peptide (C/CGRP) gene is highly expressed in C cells and in MTC [16–19]. The C/CGRP gene is also expressed in some neuron and immune cells [16, 18, 20]. However, calcitonin is [21] 1000-fold more highly expressed in C cells and MTC cells, than in these other cell types [16, 18, 20]. Thus, the calcitonin/calcitonin gene related peptide promoter (pC/CGRP) is a favorable candidate to induce specific gene expression in MTC cells for targeted therapy.

Functional elements within the full length pC/CGRP, between nucleotides -1738 and +125, include a distal tissue specific enhancer (TSE) [22, 23]; helix-loop-helix (HLH) binding sites [23–25]; a cAMP-induced enhancer (CRE) [26, 27]; the octamer motif (OCT) [25, 27]; and the core promoter, which includes a TATA box (ATAA) and the CCAAT-enhancer-binding (c/EBP) region [26]. Transgene expression in calcitonin-producing cells has been shown to be enhanced when using modified calcitonin promoters, compared to the full-length promoter [17, 21].

Recombinant adeno-associated virus (rAAV) serotype 2 is a viral vector approved by the FDA for clinical applications [28–30]. Wild-type AAV (wt AAV) is non-pathogenic [31] and is able to infect both dividing and non-dividing cells [32, 33]. It has been reported that rAAV2, with calcitonin promoter elements, can infect and transduce thyroid cells *in vitro* [21] with low efficiency. The low efficiency was likely due to use of single-stranded vectors available at the time, and Jiang et al. (2001) suggest they may have included some inhibitory elements in their promotor construct.

As a step to develop a potential novel therapy vector for MTC, with improvements over previous vectors, we engineered a double-stranded rAAV-based gene therapy vector (GTV) to express transgenes under the control of a modified, truncated (765 bp) calcitonin promoter (pM+104) [17]. We now report results from *in vitro* and *in vivo* approaches used to evaluate vectors in MTC TT cells in culture, and in a mouse xenograft tumor model system.

## Materials and methods

### Cell culture

Human MTC cell line TT (cat# CRL-1803), lung carcinoma cell line A549 (cat# CCL-185), cervical adenocarcinoma cell line HeLa (cat# CCL-2), medulloblastoma cell line Daoy (cat# HTB-186), breast adenocarcinoma cell line MCF7 (cat# HTB-22), human papillomavirus transformed kidney cell line HK-2 (cat# CRL-2190), and human embryonic kidney cell line HEK293 (cat# CRL-1573) were obtained from American Tissue Culture Collection (ATCC; Manassas, Virginia, USA). The human epidermal carcinoma cell line A431 (cat# 85090402) was obtained from Sigma-Aldrich (St. Louis, MO, USA). Human papillary thyroid carcinoma (K1, cat# 92030501) and normal thyroid epithelial (N-thy-ori 3.1, cat# 90011609) cell lines were obtained from European Collection of Cell Cultures (ECACC; Salisbury, UK). All cell lines except HK-2 cells were grown in the media recommended by each manufacturer, supplemented with 10% fetal bovine serum (Gemini Bio-Products, West Sacramento, CA, USA). HK-2 cells were grown in keratinocyte serum-free media supplemented with bovine pituitary extract and human recombinant epidermal growth factor (Life Technologies, Waltham, MA, USA).

### Plasmid construction

The full-length calcitonin promoter was amplified by polymerase chain reaction (PCR) from the MTC cell line TT using the forward 5’-CAGGGGTGTCGTGCTAAGAA-3’ and reverse 5’-CCAGAATCTCGGGGCTCACCT-3’ primers corresponding to -1738 to +125 base pairs, relative to the transcription start site. The PCR product was cloned into the pGEM vector (Promega, Madison, WI, USA) and sequenced at the University of Florida Interdisciplinary Center for Biotechnology Research (ICBR) DNA Sequencing core facility. The full-length calcitonin promoter was cloned into the pGL3 luciferase reporter vector (Promega). A pUC19 plasmid containing two copies of the modified enhancer (-1060 to -905) and one copy of the proximal promoter (-129 to +104) region were synthesized by Life Technologies and cloned into the pGL3 KpnI and NcoI sites.

Drs. George V. Aslanidi and Arun Srivastava (University of Florida) provided adeno-associated virus (AAV) plasmids with the CBA/CMV promoter/enhancer (pCBA) upstream of the GFP gene. The pCBA promoter/enhancer was removed and replaced with the pM+104 promoter/enhancer construct taken from the pGL3 plasmid (described above) using KpnI and NcoI restriction sites.

### Luciferase assays

Luciferase transfections were performed in 96-well plates with cells at 70% cell density, which is equivalent to approximately 5 x 103–5 x 10^4^ cells per well. Each well was transfected with 5 μl of serum-free media containing 0.1 μg of luciferase reporter plasmid (pGL3), 0.1 μg pRL-*renilla* (SV40 or TK promoter) control, and 0.3 μl of FuGene HD transfection reagent (Promega). At 48 hours post transfection (hpt), media was removed, then cells were briefly rinsed in phosphate-buffered saline (PBS) and lysed. All experiments were analyzed using the Dual-Luciferase Reporter Assay System (Promega) following the manufactures protocol. Luciferase activity in each well was measured at two wavelengths (530 and 640 nm) following addition of 100 μl of firefly luciferase substrate then 100 μl Stop & Glo *Renilla* substrate in a Biotek Synergy HT plate reader with Gen5 data analysis software (Biotek, Winooski, VT, USA) to detect the light output in relative luciferase units (RLU). Results are shown as the mean percentage of firefly luciferase activity (with the experimental promoter) compared to *Renilla* luciferase activity (with the internal control promoter). The averages of three independent experiments, performed in triplicate, are shown.

### AAV vector production

Recombinant AAV vectors containing a GFP gene driven by the pCBA or pM+104 promoters were generated as described [34]. Briefly, HEK293 cells were transfected with a vector genome plasmid containing inverted terminal repeats (ITR), a rep/cap plasmid and a helper plasmid, using polyethylenimine (PEI, linear, Polysciences Inc., Warrington, PA, USA). Vectors were purified 72 hpt by iodixanol gradient centrifugation (Sigma-Aldrich) and ion exchange column chromatography using HiTrap SP HP (GE Healthcare, Piscataway, NJ, USA). Viral titers were determined using nucleic acid dot blot [35].

### Recombinant AAV vector *in vitro* transduction assays

Thirty-thousand cells per well (96-well plate format) were transduced with 2 or 10 x 10^3^ vg/cell of AAV2-GFP vector, and experiments were performed in triplicate. For pixel analysis, fluorescent images were captured 48 hpi at 4x magnification using a Biotek Cytation 5 Image Reader (Biotek). Transgene expression was assessed using Image J (National Institutes of Health (NIH), Bethesda, MD, USA) to calculate the total area of green fluorescence per visual field examined for each well (pixel^2^), and for automatic counting of GFP cells using a threshold of 99.5%.

### Animal care

Animals were cared for in accordance with the principles of the Guide to the Care and Use of Experimental Animals. Five or fewer mice of the same sex were housed together in a temperature-controlled room with a 12-hour light cycle and access to standard mouse chow, bedding material, and water *ab libitum* during the entire experiment. Mice were monitored daily, for general health, weight, and tumor size. Endpoints for euthanasia (CO_2_ asphyxiation) were specified to when tumors longest diameter reached 1-cm, to minimize animals pain or distress as a result of tumor implantation. Tumor growth was measured up to several times a week, or daily near the end of the experiment, to ensure that maximal tumor size (1.2 cm) was not exceeded before the animal is euthanized. To reduce animal distress, mice were anesthetized using isoflurane (2–5%) in an inhalation chamber prior to all injection procedures.

### Animal use

All animal studies were approved by the University of Florida (IACUC) under protocol #201708136. Five-week old male and female NOD.Cg-Prkdc SCID Il2rgtm1Wjl/SzJ (NSG) mice were obtained from Jackson Laboratories (Bar Harbor, ME, USA). At 6 weeks of age, mice were injected subcutaneously in the hind limb dorsal flank with 1 x 10^7^ TT cells. When the diameter of the tumors reached ~1 cm, the rAAV2 virus was injected directly into the tumor along with a rhodamine dye (rhodamine B isothiocyanate- Dextran, Sigma-Aldrich). Forty-eight or 72 (when indicated) hours after injection, the mice were euthanized on GD 18 by CO_2_ asphyxiation followed by cervical dislocation and whole blood collection by terminal cardiac puncture, and their tumors and livers were harvested and processed for analysis.

### Quantitative PCR of vector nucleic acid isolated from tumor tissue

At the time of tissue harvest, a slice of ~ 30–50 mg tumor tissue was excised from the part of tumor bearing the traces of the rhodamine using a razor blade, and preserved along with liver lobes from the same mice at -20°C with RNAlater solution (Ambion, Austin, TX, USA) for future processing. Tissue samples stored in RNALater solution (Ambion) were used for RNA and DNA purification with RNeasy Plus and DNeasy Blood & Tissue kits (QIAGEN, Germantown, MD, USA) correspondingly. Samples were finely minced on an ice chilled block by scalpel blades and split equally for RNA and DNA isolation. Complementary DNA (cDNA) was synthesized using iScript Reverse Transcription Supermix (Bio-Rad Laboratories, Hercules, CA, USA) with an RT cycle profile: 25°C, 5 min; 46°C, 20 min; 95°C, 1 min; 4°C hold. Real-time PCR (qPCR) was conducted using SsoAdvanced^™^ Universal SYBR® Green Supermix (Bio-Rad Laboratories) and primers, targeting amplicon of about 120 bp: CommonGFP_For: 5'- TGCTTGTCGGCCATGAT -3' and CommonGFP_Rev2: 5'- AACTACAAGACCCGCGC -3'. PCR conditions were set according to the manufacturer of the real-time PCR amplification device (Bio-Rad CFX96), protocol CFX2stepAmp.prcl: 95°C, 2 minutes; 2 Step PCR, 39 cycles: 95°C,10 seconds, 55°C, 30 seconds; Melting Curve Analysis. For quantitative analysis, standard dilutions of plasmid DNA samples were used that included the same GFP gene as in rAAV vectors.

### Immunohistochemistry

Tumors were sliced in half along a plane that aligned perpendicular to the approximate injection site. Following fixation with 4% paraformaldehyde (PFA), samples were embedded in paraffin and sectioned with a Micron HM325 microtome (Walldorf, Germany). Slides containing 5 μm thick sections were deparaffinized and rehydrated through a series of ethanol (EtOH) /water washes. Following a 95°C 25-minute antigen retrieval in Trilogy unmasking solution (Cell Marque, Rocklin, CA, USA). Slides were serum treated for one hour at room temperature with 2% normal goat serum (Vector Laboratories, Burlingame, CA, USA). Chicken antibody to GFP (Abcam, Cambridge, MA, USA) was applied at 1:750 overnight at 4°C. Positive staining was detected with a one-hour incubation in 1:500 Alexafluor goat anti-chicken 488 (Invitrogen, Carlsbad, CA, USA) mounted in Vectashield (Vector Laboratories) with 4’, 6-diamidino-2-phenylindole (DAPI) as counterstain.

Immunohistochemical staining was performed using a Rabbit anti-Calcitonin (1:250; Abcam) antibody as previously described [36]. Images were captured on a Zeiss Axio Vert.A1 inverted light microscope (Zeiss, Thornwood, NY, USA).

### Histology

The tumor and liver tissues were removed from the mice, cut in half, and placed inside of 20ml scintillation vials. The tissues were immersion fixed overnight in 4% paraformaldehyde (PFA) before rinsing with 1x phosphate buffered solution (PBS) and transferred to a 25% sucrose solution to equilibrate for three days at 4°C. Once equilibrated, prepared tissues were embedded in an optimal cutting temperature (OCT) medium, and frozen in a 2-methylbutane/dry ice slurry. The sections were cut at 7μm thickness, thaw mounted onto positively charged slides and cover slipped in Vectashield mounting media containing DAPI (Vector Laboratories). GFP and DAPI in tissue sections were visualized using the Olympus BX43 upright microscope and DP80 camera mount, using Olympus cellSens software (Olympus, Pittsburgh, PA, USA).

For processing in hematoxylin and eosin (H&E), the frozen tumors were cut into 7μm thick sections, thaw mounted onto positively charged slides. Slides were rehydrated to water through a graded ethanol series (100% EtOH twice for 2 minutes each, 95% EtOH for 3 minutes, 70% EtOH for 1 minute, and water twice for 1 minute each). In the staining process, the slide was placed in hematoxylin for 1 minute and 30 seconds, rinsed in water for 15 seconds then 1 minute, placed into a defining solution for 45 seconds, water for 1 minute, bluing solution for 1 minute, water for 1 minute, 80% EtOH for 1 minute, Eosin for 1 minute, and 100% EtOH three times for 1 minute each. The slide was then mounted in Vectashield with DAPI as a counterstain (Vector Laboratories) and examined on a Leica DM2500 microscope and Retiga 4000R camera mount, using Q imaging software (Leica Microsystems, Morrisville, NC, USA).

### Quantitative analysis of tumor images

To quantify GFP intensity in data images of tumor central sections, we used ImageJ to measure the total area of GFP pixels (mean, gray value, and standard deviation) under a limited threshold of approximately 99.5%. Two to five images per central section were averaged, per tumor. The average GFP pixels^2^ per image for uninfected tissue images was subtracted from mean values for rAAV infected tissue images. GFP-expressing cells were auto picked and counted using ImageJ software [37]. Data images were converted to 8-bit and a Gaussian Blur (sigma = 3) was applied. The Bernsen Auto Local Threshold counting method (using the following parameters: radius = 8 pixels, parameter_1 = 30) was used [38, 39]. GFP cells larger than 155 pixel^2^, with a circularity between 0.4 and 1.0, and DAPI-stained nuclei larger than 95 pixel^2^ with a circularity between 0.1 and 1.0, were counted.

### Statistical analysis

Values reported are means, and error bars represent the standard error of the mean (SEM). Pairwise differences between means were evaluated using Student’s T-test, and resulting p-values are reported. Two-tailed T-tests were used, except when comparing treatments that differed in dose or treatment duration, for which one-tailed T-tests were used. One-way ANOVA was used for multiple comparisons followed by *post hoc* pairwise T-tests. T-test p-values < 0.05 were considered statistically significant and are indicated in the figures with asterisks using the following convention: * = < 0.05, ** = < 0.01, *** = < 0.001. Circled asterisks follow the same convention, but indicate both significant and Bonferroni corrected p-values (p_c_-values), corrected for multiple comparisons. Statistical analyses were done using Microsoft Excel V.16.16.15 (2018) and GraphPad Prism V.7 software (La Jolla, California, USA).

## Results

### Calcitonin promoter-mediated luciferase gene expression

**Fig 1:**
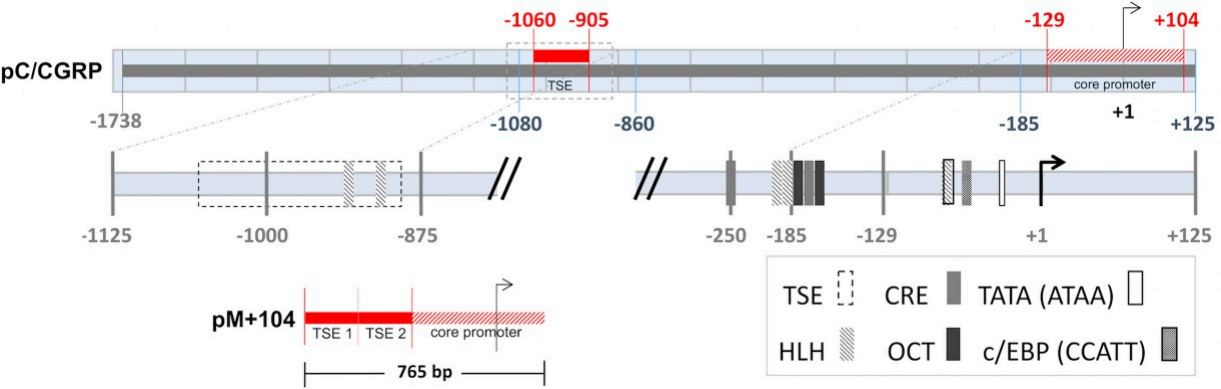
Schematic representation of the calcitonin/calcitonin gene-related peptide promoter (pC/CGRP) and *cis*-regulatory elements. Functional elements within the full length pC/CGRP, between nucleotides -1738 and +125, include a distal tissue specific enhancer (TSE) [22, 23]; helix-loop-helix (HLH) binding sites [23–25]; cAMP-induced enhancer (CRE) [26, 27]; the octamer motif (OCT) [25, 27]; and the core promoter, which includes a TATA box (ATAA) and the CCAAT-enhancer-binding (c/EBP) region [26]. The mini promoter pM+104 (765 bp in length) was constructed by fusing two copies of the C/CGRP TSE (-1060 to -905), TSE 1 and TSE 2, with one copy of the C/CGRP core promoter (-129 to +104), as previously described in Messina et al., 2000 [17].

**Fig 2:**
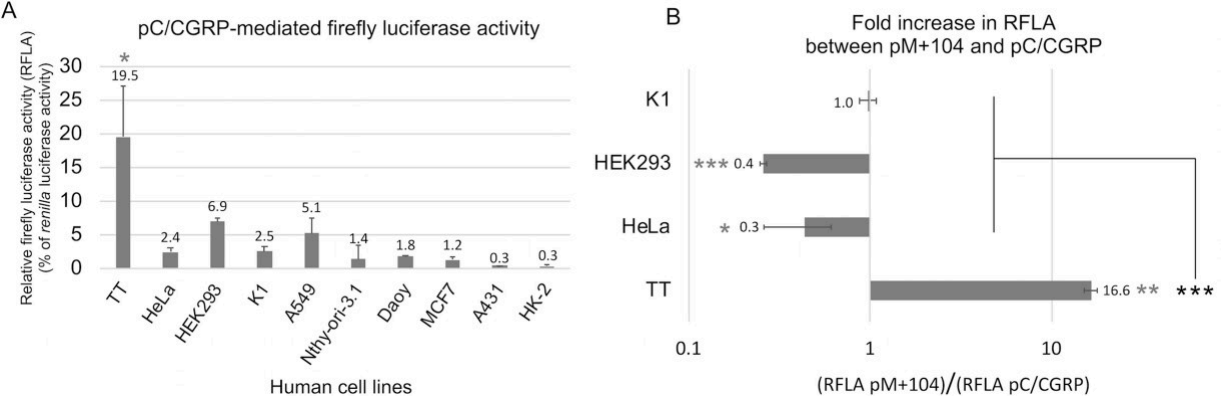
Calcitonin promoter mediates cell-specific gene expression in the TT MTC cell line. Relative firefly luciferase activity (RFLA) was determined from double transfection experiments using two luciferase gene (*firefly* and *renilla*) reporter plasmids. RFLA is shown as a percentage of *Renilla* luciferase activity. (A) RFLA from pLG3 with the full-length pC/CGRP (-1738 to +125) in MTC cell line TT and nine non-MTC cell lines, are shown. Circled asterisk indicates a significant (p_c_ < 0.05) difference in RFLA between TT and all cell types tested. (B) RFLA from pGL3 with the full-length pC/CGRP was compared to pGL3 with the truncated pM+104 promoter. Changes in expression levels are show as the fold-increase of luciferase activity observed using pM+104 over using pC/CGRP for TT, HeLa, HEK293 and K1 cell lines. Asterisks indicate a significant difference from pairwise two-tailed T-test between pM+104 and pC/CGRP, for an indicated cell type (* = p < 0.05, *** = p < 0.001). Circled asterisks indicate a significant difference between TT and all cell types tested, in the amount of change in expression levels (*** = p_c_ < 0.001).

As a step toward developing a potential novel therapy vector with tissue specific expression in MTC cells, we utilized key elements of the calcitonin promoter. We modified the pGL3 *firefly* luciferase gene reporter plasmid to include the full-length pC/CGRP (Fig 1). Using transient transfection with pGL3 plasmids, we tested for luciferase gene expression in MTC and non-MTC human cell lines. Co-transfection experiments of pGL3-pC/CGRP with *renilla* luciferase gene control vectors pRL-SV40 were used to measure the relative firefly luciferase activity (RFLA) in human cell lines. The MTC-derived cell line TT transfected with pGL3-pC/CGRP yielded a RFLA (19.5% +/- 7.5 of *Renilla* luciferase activity), which was significantly (p_c_ < 0.05) higher than nine non-MTC cell lines tested, including cell lines derived from human follicular thyroid tissue, K1 (2.48% +/- 0.78) and Nthy-ori-3.1 (1.4% +/- 1.95) (Fig 2A).

The pGL3 plasmid was modified to include the mini promoter pM+104. Transient transfection experiments comparing the RFLA from plasmid pGL3-pC/CGRP with that from pGL3-M+104 (Fig 2B) demonstrated a significant increase (16.6-fold, p < 0.0036) in RFLA in TT cells, significant reductions in RFLA in HeLa and HEK293 cells (2.3-fold, p = 0.0196, and 3.9-fold, p = 0.0001, respectively), and no significant change in K1 cells. Furthermore, the TT cell line demonstrated a significantly (p_c_ < 0.001) higher change in RFLA from pC/CGRP to pM+104, than all other cell types tested (Fig 2B).

These data show that pC/CGRP confers MTC cell-specific gene expression, and the pM+104 mini promoter improves that specificity.

### *In vitro* transgene expression of rAAV-pM+104-GFP vectors in the MTC TT cell line

**Fig 3:**
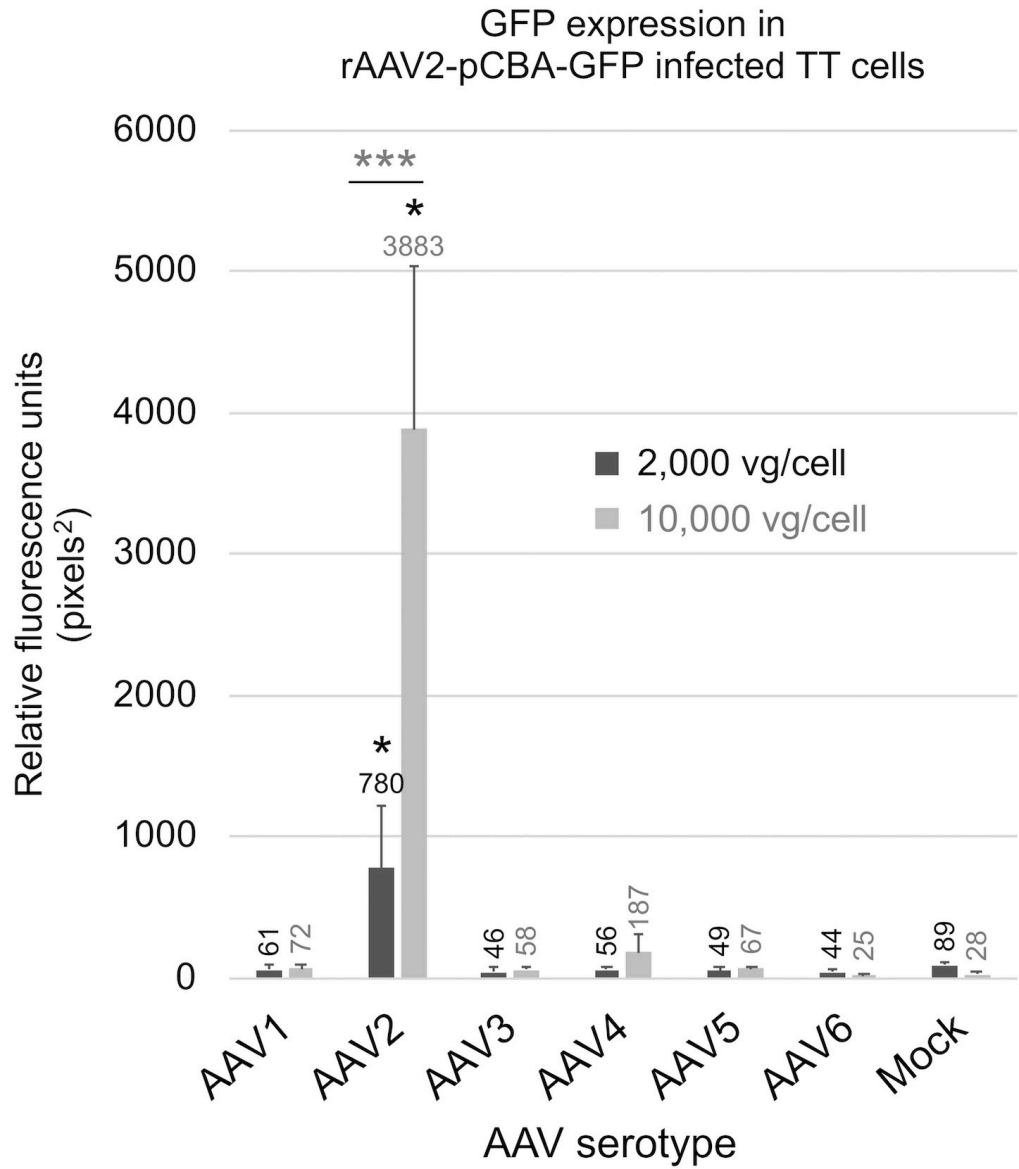
rAAV serotype 2 viral vector promotes gene expression in MTC cell line TT. GFP expression was quantified as relative fluorescence (GFP pixels^2^) in TT cells treated using an rAAV2-pCBA-GFP vector (a non-tissue specific vector) with capsid serotypes one through six. Two infection dose levels were tested, 2,000 vg/cell and 10,000 vg/cell. Asterisks indicate a significant (** = p < 0.01) difference in GFP expression, when comparing the two dose levels for AAV serotype 2, using a one-tailed T-test. The circled asterisk indicates a significant (* = p_c_ < 0.05) increase in GFP expression at the higher dose level (10,000 vg/cell), when comparing AAV2 with to all other serotypes tested, using pairwise one-tailed T-tests, following ANOVA.

Next, we identified which rAAV serotypes show high levels of transgene expression in TT cells. First, rAAV serotypes 1–6, each with the chicken beta promoter and cytomegalovirus enhancer control region (pCBA) upstream of the GFP gene, were tested to determine which serotype best promoted transgene expression in MTC TT cell line. AAV2 was the only serotype to give GFP expression significantly higher (p < 0.01) using higher vector titer (10,000 vg/cell) than with infections at lower titer (2,000 vg/cell) (Fig 3). The rAAV2-pCBA-GFP vector infected at high titer (10,000 vg/cell) produced significantly (p_c_ < 0.05) higher RFLA than the other serotypes tested (Fig 3). Thus, the rAAV2 capsid form was selected for use with pM+104-GFP vector genomes.

**Fig 4:**
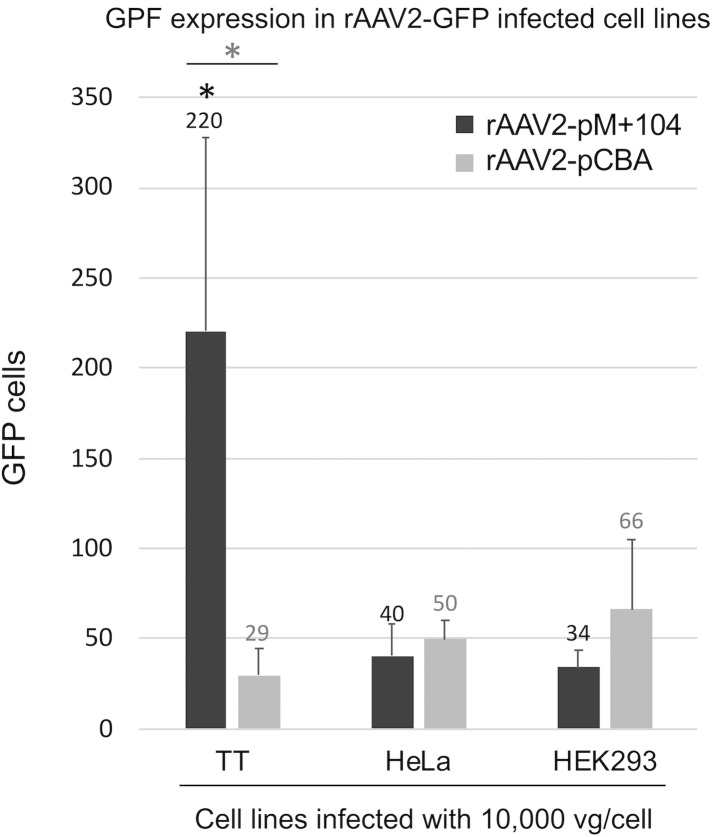
rAAV2-pM+104 GFP vector improves GFP expression in MTC cell line TT, *in vitro*. Images of cultured cells (MTC TT, HeLa, and HEK293) infected at an MOI of 10,000 vg/cell with the non-tissue specific control vector rAAV2-pCBA-GFP, and with test vector rAAV2-pM+104-GFP were analyzed. GFP cells were automatically counted using ImageJ, and the two vectors were compared in a given cell type, using two-tailed T-tests. Asterisk indicates significant (p = 0.0126) difference between the non-tissue specific control vector (rAAV2-pCBA-GFP) and the test vector (rAAV2-pM+104-GFP) in TT cells. Asterisk in a circle indicates a significant (* = p_c_ < 0.05) difference between TT and all cell types tested, in the number of GFP cells detected following infection with rAAV2-pM+104-GFP. Comparisons were made using pairwise two-tailed T-tests, following ANOVA.

The rAAV vector genome plasmid pdsAAV2-pCBA-GFP, with a double stranded vector genome, was next modified by removing pCBA and replacing it with pM+104. The resulting pdsAAV2-pM+104-GFP plasmid also contained flanking rAAV2 inverted terminal repeats (ITRs) and a GFP gene. This construct was used to produce vectors with the wt rAAV2 capsid encasing the ITR-pM+104-GFP-ITR vector genome. rAAV2-pM+104-GFP and rAAV2-pCBA-GFP vectors were each tested *in vitro* for GFP protein expression in TT, HeLa, and HEK293 cells. TT cells infected with rAAV2-pM+104-GFP showed a significant (p_c_ < 0.05) increase in the number of GFP expressing cells detected per region of interest (220.4 +/- 107.7) over TT cells infected with rAAV-pCBA-GFP (29.4 +/- 15.3), HeLa cells infected with either pM+104 (40.1 +/- 17.7) or pCBA (49.7 +/- 10.0) GFP vectors, and HEK293 cells infected with either pM+104 (33.9 +/- 9.0) or pCBA (66.2 +/- 38.8) GFP vectors, (Fig 4). rAAV-pCBA-GFP did not yield significant differences in the number of GFP-expressing cells detected between cell types.

These data demonstrate that the AAV2 serotype (among AAV1-6) targets MTC cells. Further, the rAAV2-pM+104-GFP vector improves MTC cell-specific gene expression over the control promoter (pCBA), *in vitro*.

### *In vivo* transgene expression of rAAV-pM+104-GFP vectors in MTC xenograft tumors

**Fig 5:**
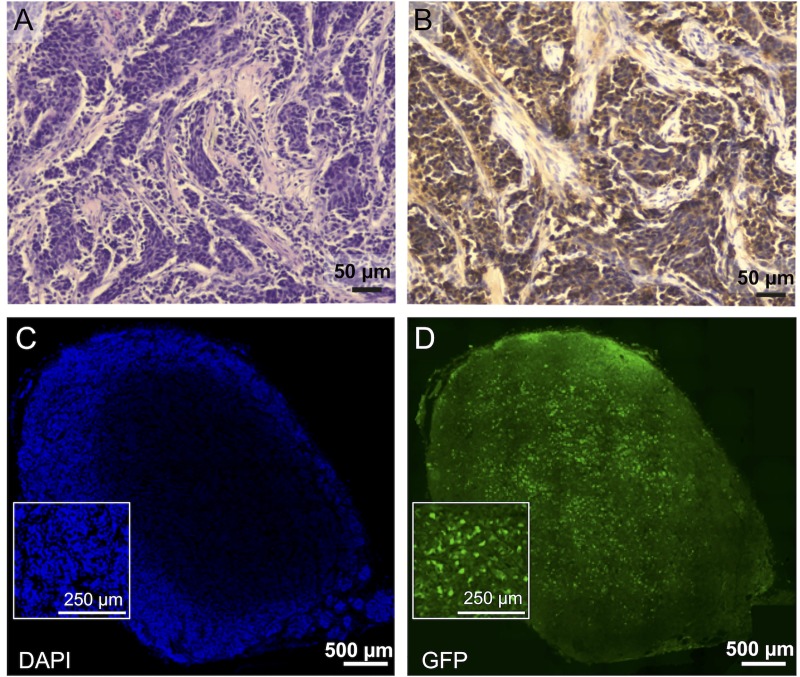
Histological analysis of rAAV GFP infected TT cell xenograft tumors. (A) H&E staining demonstrates MTC morphology and vascularization in xenograft tumors. (B) Strong positive immunohistochemical staining (brown) of xenograft tumor tissue is shown for endogenously expressed calcitonin, a hallmark of C cells and MTC. (C and D) Representative fluorescence microscopy images of the central section of a whole tumor that was infected with 2 x 10^10^ vg/tumor of rAAV2-pM+104-GFP for 48 hours. (C) DAPI staining and (D) GFP expression were observed in infected tumor tissue. Scale bars are indicated.

The rAAV2-GFP vectors were next tested in a TT xenograft mouse model system to determine if the MTC could be targeted *in vivo*. Tumors were induced through subcutaneous injection of TT cells into the dorsal flank of six-week old male and female NOD.Cg-Prkdc SCID Il2rgtm1Wjl/SzJ (NSG) mice. About 8 weeks after tumor initiation, tumors reached about 1 cm in diameter and were infected via direct injection. Tumors infected with 2 x 10^10^ vg/tumor of rAAV2-pM+104-GFP for 48 hours. Infected tumor tissue showed normal MTC tumor morphology and vascularization, calcitonin staining (a hallmark for C-cells and MTC), and GFP expression (Fig 5).

**Fig 6:**
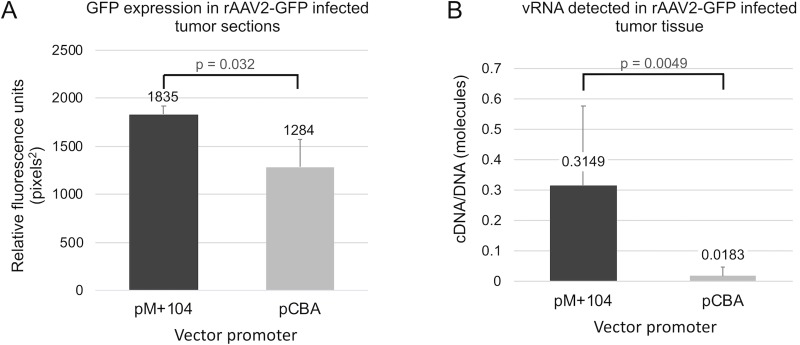
Quantification of rAAV2-GFP transduction efficiency in TT cell xenograft tumors. One-cm tumors were infected with 2 x 10^10^ vg/tumor of rAAV2-pM+104-GFP for 48 hours, and then the tumor tissue was analyzed for *gfp* gene expression. (A) Images of infected tumor tissue sections were analyzed using ImageJ, for GFP intensity, reported as relative fluorescence units (RFU) in pixels^2^. N = 3 (pCBA) and 4 (pM+104) tumors. (B) Vector nucleic acid content in rAAV-infected tumor tissue was quantified using qPCR. vRNA quantities normalized to vDNA content are shown. P values < 0.05 are indicated and were considered significant in the indicated pairwise comparisons, using two-tailed T test analysis. N = 6 rAAV2-pCBA-GFP infected tumors, and N = 9 rAAV2-pM+104-GFP infected tumors.

To compare pM+104 to pCBA for GFP expression in our TT xenograft mouse model, 1-cm tumors were injected with 2 x 10^10^ vg of either rAAV2-pM+104-GFP or rAAV2-pCBA-GFP, and then tumors were processed for GFP expression analysis 48 hours later. Tumors injected with rAAV2-pM+104-GFP showed significantly (p = 0.018) higher GFP intensity (1835.2 +/- 86.4 pixels^2^) than tumors injected with rAAV2-pCBA-GFP (1284.4 +/- 282.3 pixels^2^) (Fig 6A). Tumors treated with pM+104-GFP vector were found to contain 22.9 times more (p = 0.0097) vRNA molecules per vector genome detected than pCBA vector-treated tumor tissue (0.3149 +/- 0.2642, and 0.0183 +/- 0.0309) (Fig 6B). Vector nucleic acid was not detected off-target in the liver tissue harvested from vector treated animals.

These data demonstrate that rAAV2-pM+104 vectors can promote transgene expression in MTC xenograft tumors, and support the hypothesis that pM+104 promotes higher levels of *gfp* gene and GFP protein expression in MTC TT tumor cells than with the ubiquitously expressed positive control vector, rAAV-pCBA.

### Dose and treatment duration responses in rAAV2-pM+104-GFP vector-transduced MTC xenograft tumors

**Fig 7:**
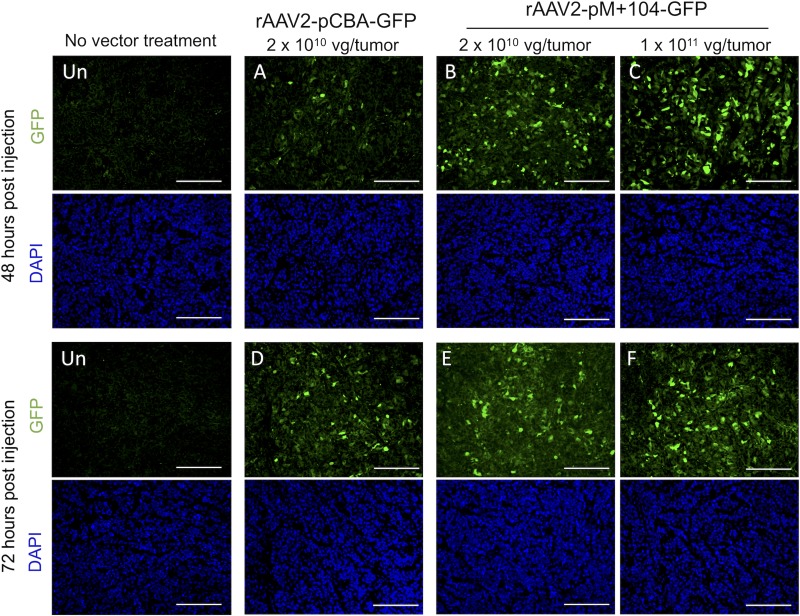
Visualization of rAAV2-GFP transduced TT cell xenograft tumor tissue. One-cm tumors were injected with rAAV2-pCBA-GFP vector (2 x 10^10^ vg/tumor) or rAAV2-pM+104-GFP vector (2 x 10^10^ or 1 x 10^11^ vg/tumor), for 48 or 72 hours prior to tumor removal. Tumor tissue sections were analyzed for GFP expression (green) and DAPI staining (blue) using fluorescence microscopy and image analysis. Representative GFP and DAPI images from uninfected (Un) tissue, and tissue infected with vector (A-F) are shown. Tumor treatments (A-F) varied in vector, dose, and infection duration. Scale bars are 250 *μ*m.

**Fig 8:**
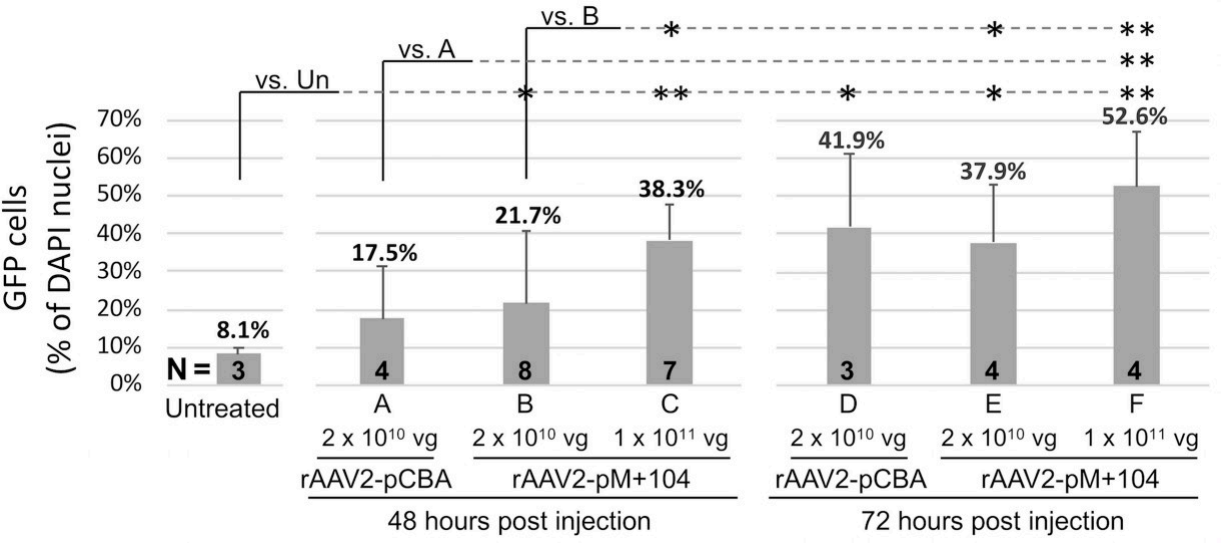
Quantification of dose- and duration-dependent improvement in transduction efficiency by rAAV-GFP in TT cell xenograft tumor. GFP-expressing cells, detected in fluorescence micrographs of tumor sections, were counted automatically using Image J, and reported as a percentage of DAPI nuclei. Images from uninfected (Un) tumor sections, and vector-infected tumor sections (A-F) were analyzed. Tumor treatments A-F varied in vector, dose, and infection duration. P-values < 0.05 were considered significant, as determined by T-test for pairwise comparison of treatments, following ANOVA, and are indicated by asterisks. Circled asterisks indicate p-values that were Bonferroni corrected for multiple comparisons. Corrected p-values (p_c_) < 0.05, were considered significant for the indicated pairwise comparisons between treatments. The number of tumors (N) per treatment are shown in bolded integers.

To determine the impact of vector amount (dose) and treatment exposure time (duration) on rAAV2-M+104-GFP vector transduction efficiency in MTC TT xenograft tumors, we tested several different vector treatments (A-F, in Figs 7 and 8), varying the injected vector dose (2 x 10^10^ vg/tumor or 1 x 10^11^ vg/tumor), and the infection duration (48 hours or 72 hours) following vector injection. Transduction efficiency was measured by counting detectible GFP-expressing tumor cells (green cells in Fig 7). GFP cell counts are reported as percentages of DAPI stained nuclei (Fig 8).

Nearly all vector treatments, yielded GFP tumor cell counts significantly (p < 0.05) higher than the background detected in uninfected (un) tumor tissue (8.5% +/- 1.9), including treatment B, rAAV2-pM+104-GFP at 2 x 10^10^ vg/tumor for 48 hours (21.7% +/- 9.5, p = 0.0205); treatment C, rAAV2-pCBA-GFP at 1 x 10^11^ vg/tumor for 48 hours, 38.3% +/- 15.5, p = 0.0058 (p_c_ = 0.0403)); treatment D, rAAV2-pM+104-GFP at 2 x 10^10^ vg/tumor for 72 hours (41.9% +/- 19.0, p = 0.0187), treatment E, rAAV2-pM+104-GFP at 2 x 10^10^ vg/tumor for 72 hours (37.9% +/- 15.5, p = 0.0104), and treatment F, rAAV2-pM+104-GFP at 1 x 10^11^ vg/tumor for 72 hours (52.6% +/- 14.2, p = 0.0006 (p_c_ = 0.0039)). Treatment A, rAAV2-pCBA-GFP with 2 x 10^10^ vg for 48-hours duration, yielded 17.5% +/- 14.0 GFP cells, which was not significantly (p = 0.1553) higher than that detected in uninfected tissue (Fig 8).

Pairwise comparisons with rAAV2-pM+104-GFP treatment variants, showed that treatment B (2 x 10^10^ vg/tumor for 48 hours) yielded significantly fewer (p < 0.05) GFP cells detected than all rAAV2-pM+104-GFP treatments with a higher dose (treatment C, at 1 x 10^11^ vg/tumor for 48 hours, p = 0.0122) or a longer duration (treatment E, 2 x 10^10^ vg/tumor for 72 hours, p = 0.0218), or both higher dose and duration (treatment F, 1 x 10^11^ vg/tumor for 72 hours, p = 0.006 (p_c_ = 0.0039)) (Fig 8). For pairwise comparisons between rAAV2-pM+104-GFP and rAAV2-CBA-GFP, treatments did not differ with statistical significance (p > 0.05), except treatment F yielded significantly higher GFP cells detected than treatment A (p– 0.0063 (p_c_ = 0.0438) (Fig 8). No significant difference was detected by pairwise comparison between treatments with a 72-hour durations (treatments D, E, and F).

These data show dose- and infection-duration dependencies on transduction efficiency with rAAV2-pM+104 vectors in MTC tumor cells. The greatest number of GFP cells were detected with a 1 x 10^11^ vg dose for 72 hours (52.6% +/- 14.2), suggesting that even higher dosages and longer infection durations may improve transduction in tumors.

## Discussion

We report a novel rAAV vector (rAAV2-pM+104-GFP) with increased transgene expression in the MTC cell line TT over the non-MTC cell lines tested, demonstrating tissue specification. We are able to use and rAAV2-GFP vector with a miniature (~765 bp) modified calcitonin promoter/enhancer construct (pM+104) to give strong and specific expression in MTC cells *in vitro* and *in vivo*. The small size of this modified calcitonin promoter, compared to the full calcitonin promoter region, facilitates packaging of larger therapeutic genes in an rAAV vector.

We predict that rAAV2 serotype vectors showed higher transduction efficiency in TT cells over all other serotypes tested (AAV1-6) due to the cell-surface receptors displayed on TT cells. For example, the combination of heparan sulfate proteoglycan (HSPG) as a primary receptor, with ***α***V*β*5 integrin as a co-receptor is unique for AAV2 cell entry and transduction, not shared by the other serotypes tested [40] In a related study (data not shown), we ablated the known rAAV2 heparin binding site [41, 42] through capsid mutagenesis and abolished rAAV-pM+104-GFP vector transduction in TT cells.

In these studies, we have demonstrated robust dose and duration-dependent GFP expression efficiency with rAAV-pM+104-GFP vector in MTC tumor xenografts, revealing the potential for further optimization of vector administration to improve the expression of therapeutic genes in tumor tissue. We observed over 52.6% of tumor cells expressing GFP in rAAV-pM+104-GFP-treated (1 x 10^11^ vg per tumor) tumor tissue at 72 hours. This relatively low percentage of GFP cell, though, may be due to the fact that MTC tumor cells can be multinucleated, and cells that express lower levels of GFP, below our threshold of detection, are not counted.

A previous report examining rAAV transduction in TT cells showed 40% infection efficiency *in vitro*, but with adenovirus type 5 (Ad-5) co-infection [21]. In our studies we administer rAAV alone (without the rAAV infectivity boost that comes with Ad co-infection), and *in vitro* and *in vivo*, thus demonstrating improved infectivity of the vector with better clinical relevance (as Ad is not approved for use in gene therapy), and we have expanded the model into animal studies.

Although rAAV2 has been introduced in the clinic, and is a favorable serotype for preclinical studies in mice, it is not a favored serotype to treat diseases in regions of the body that are outside of the central nervous system (CNS), and that do not have immune privilege. Strategies have been proposed to overcome rAAV2 neutralizing antibodies (Nab), which can limit the transduction efficiency of therapeutic vectors, including using high vector doses, pre-dosing with empty capsids to saturate the Nab, and suppressing the B cell response prior to drug administration [43]. Other rAAV serotypes and vector modifications will need to be tested for transducing MTC cells in humans. Furthermore, additional pre-clinical studies are needed using the mouse xenograft MTC model described in this report, but with systemic (tail vein) vector administration. Off-target infection using the GFP vectors described in this study, would be detected as GFP expression in non-MTC cells, and non-tumor tissues. Such studies will allow us to determine if vectors can target MTC cells *in vivo* and to assess vectors for off-target infection, as AAV2 is known to infect neurons, which express C/CGRP at low levels. AAV capsid peptide inserts, using peptides know to target MTC, may be useful to further narrow vector specificity to MTC [44, 45].

Multiple strategies for gene therapy have been proposed for treatment of thyroid carcinomas including targeting of specific pathways, reintroduction of the sodium iodine symporter, immune modulation, and gene-directed enzyme/prodrug therapy (GDEPT) [46]. As the RET gene is commonly mutated in both hereditary and sporadic MTCs, drug treatment inhibiting the RET pathway has been used [47–49]. Improved, specific methods of inhibiting RET could be delivered using rAAV vectors. Many GDEPT methods not only induce cell death in targeted cells, but result in bystander killing of surrounding cells due to the release of toxic metabolites [20]. The rAAV2-pM+104 vector reported here transduced up to 52.6% of TT cells *in vivo*, thus a GDEPT method, as well as multiple injections per tumor and increased vector dosage may be beneficial in targeting tumors without 100% transduction efficiency.

In conclusion, we report a novel rAAV vector for *in vivo* transgene expression in MTC. Strong and specific expression was achieved in MTC cells due to the use of the modified calcitonin promoter used in our rAAV2 vectors, and continued expression of calcitonin in medullary carcinomas was confirmed [50, 51]. We speculate that a gene therapy that utilizes the calcitonin promoter has the potential to treat MTC across clinical subtypes. With further modification, and the introduction of therapeutic genes, rAAV vectors could emerge as a useful tool in targeting and treating of MTC.
